# Potential for Occupational Exposure to Engineered Carbon-Based Nanomaterials in Environmental Laboratory Studies

**DOI:** 10.1289/ehp.0901076

**Published:** 2009-09-23

**Authors:** David R. Johnson, Mark M. Methner, Alan J. Kennedy, Jeffery A. Steevens

**Affiliations:** 1 U.S. Army Engineer Research and Development Center, Environmental Laboratory, Vicksburg, Mississippi, USA;; 2 National Institute for Occupational Safety and Health, Nanotechnology Research Center, Cincinnati, Ohio, USA

**Keywords:** aerosolization, ecotoxicology, multiwalled carbon nanotubes, nanomaterials, occupational exposure, sonication

## Abstract

**Background:**

The potential exists for laboratory personnel to be exposed to engineered carbon-based nanomaterials (CNMs) in studies aimed at producing conditions similar to those found in natural surface waters [e.g., presence of natural organic matter (NOM)].

**Objective:**

The goal of this preliminary investigation was to assess the release of CNMs into the laboratory atmosphere during handling and sonication into environmentally relevant matrices.

**Methods:**

We measured fullerenes (C60), underivatized multiwalled carbon nanotubes (raw MWCNT), hydroxylated MWCNT (MWCNT-OH), and carbon black (CB) in air as the nanomaterials were weighed, transferred to beakers filled with reconstituted freshwater, and sonicated in deionized water and reconstituted freshwater with and without NOM. Airborne nanomaterials emitted during processing were quantified using two hand-held particle counters that measure total particle number concentration per volume of air within the nanometer range (10–1,000 nm) and six specific size ranges (300–10,000 nm). Particle size and morphology were determined by transmission electron microscopy of air sample filters.

**Discussion:**

After correcting for background particle number concentrations, it was evident that increases in airborne particle number concentrations occurred for each nanomaterial except CB during weighing, with airborne particle number concentrations inversely related to particle size. Sonicating nanomaterial-spiked water resulted in increased airborne nanomaterials, most notably for MWCNT-OH in water with NOM and for CB.

**Conclusion:**

Engineered nanomaterials can become airborne when mixed in solution by sonication, especially when nanomaterials are functionalized or in water containing NOM. This finding indicates that laboratory workers may be at increased risk of exposure to engineered nanomaterials.

Large amounts of engineered nanomaterials are generated annually, and each possesses its own unique characteristics. Much work has focused on carbon-based nanomaterials (CNMs), such as fullerenes and carbon nanotubes, because of their strength, conductivity, and applicability for biomedical applications (Ajayan and Zhou 2001). Consequently, considerable effort has been dedicated to understanding the health effects of these nanomaterials before they are widely used in consumer products where the potential for exposure to the general public would be increased (Helland et al. 2007). This is a proactive approach that has not been applied to some other classes of chemicals in the past, such as asbestos. Environmental researchers are actively examining the fate and effects on CNMs in environmentally relevant systems (Asharani et al. 2008; Farré et al. 2009; Helland et al. 2007; Kennedy et al. 2008; Klaine et al. 2008). Furthermore, researchers at the National Institute for Occupational Safety and Health (NIOSH) and other agencies are examining the potential occupational exposures to, and respiratory effects of, CNMs (Han et al. 2008; Helland et al. 2007; Maynard et al. 2004; Methner 2008).

Currently, no occupational exposure limits govern workplace exposure to engineered nanomaterials (Methner 2008). NIOSH recommends using basic safety requirements when handling dried CNMs and other nanomaterials (NIOSH 2009). Less attention has been devoted to workplace exposure and safety of engineered nanomaterials in liquid suspensions. CNMs and other nanomaterials are usually placed into liquid suspension for easier delivery to experimental models. Conventional wisdom suggests that nanomaterials in liquid suspension generally pose lower inhalation risk to workers. However, CNMs and other nanomaterials often agglomerate in aqueous suspension, requiring continuous mixing or sonication to deagglomerate nanomaterials. It is possible that this common laboratory process results in the release and dispersion of nanomaterials into the air via small water droplets. This may concern scientists in general, but especially ecotoxicologists, environmental scientists, and environmental engineers working with nanomaterials in simulated natural waters. These researchers routinely generate environmentally relevant matrices in the laboratory, including waters with natural organic matter (NOM), which acts as a surfactant that enhances the stability of nanoparticle dispersions (Hyung et al. 2007; Hyung and Kim 2008; Kennedy et al. 2008; Lin and Xing 2008; Saleh et al. 2008; Xie et al. 2008). Thus, sonication of NOM-containing water can, in theory, result in increased aerosolization of the engineered nanomaterials when compared with the same material sonicated in deionized (DI) water.

It was with this premise that researchers at the U.S. Army Engineer Research and Development Center’s Environmental Laboratory (ERDC-EL) volunteered to be part of a nationwide field study of potential occupational exposure to nanomaterials, currently being conducted by the NIOSH Nanotechnology Research Center (NTRC). The specific research objective of the present study was to investigate the potential for the release of airborne CNMs due to research involving the handling and mixing of CNMs with environmentally relevant matrices. The NIOSH NTRC field research team evaluated two laboratory processes: *a*) transfer of CNMs from storage containers to a weighing balance, and *b*) sonication. We used both quantitative and qualitative methodologies in this range-finding study to determine the presence and concentrations of airborne nanoparticles.

## Materials and Methods

### Chemicals

We purchased fullerenes (≥ 99.5% purity) from SES Research (Houston, TX). Raw multiwalled carbon nanotubes (MWCNT) (outer diameter, 10–20 nm; length, 10–30 μm; > 95% purity) and functionalized MWCNT (i.e., hydroxylated; MWCNT-OH) (outer diameter, 20–30 nm; length, 10–30 μm; > 95% purity) were purchased from Cheap Tubes, Inc. (Brattleboro, VT). Carbon black (CB; amorphous carbon, average primary particle size of 15 nm) from Printex 95 was purchased from Evonik North America (formerly Degussa; Parsippany, NJ). NOM from the Suwannee River was purchased from the International Humic Substance Society (Atlanta, GA).

### Laboratory processes evaluated

**Figure 1:**
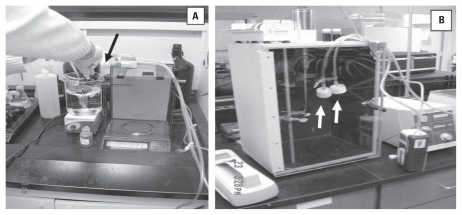
Experimental setup for evaluating engineered carbon-based nanomaterial in the laboratory using air filters to collect airborne CNM for TEM analysis. (*A*) Weighing CNMs in an electronic balance and transferring CNMs to a beaker of water being stirred; this process occurred inside a hood with no ventilation. (*B*) Sonication process inside an unventilated enclosure. In both *A* and *B*, note the proximity of the air filter (arrows) to the laboratory processes.

The first laboratory process (Figure 1A) we evaluated was weighing 4–200 mg of each of the different CNMs on an electronic balance and transferring the CNMs to a beaker of water stirring atop a Corning magnetic mixing plate (Cole-Palmer, Vernon Hills, IL). This procedure was performed inside a laboratory safety hood with the air flow turned off temporarily and the sash halfway open. This was done because the hood air velocity (measured at 100 ft/min at the face) was high enough to result in loss of nanomaterial from the spatula during the transfer from the material container to the analytical balance. The second laboratory process (Figure 1B) we evaluated was probe sonication (50 W; 40% duty cycle) of 100 mg/L previously mixed CNMs for 20 min inside an unventilated sonication enclosure (Branson Sonifier model 450; Branson Ultrasonic, Danbury, CT). CNMs were sonicated in DI water or hard reconstituted water with and without 100 mg/L NOM. Personal protective equipment worn by workers when performing weighing and transfer tasks and sonication processes consisted of a cotton laboratory coat, latex gloves, and an N95 filtering facepiece respirator.

### Airborne particle detection

We used two direct-reading, real-time instruments to determine whether CNM emissions occurred during these laboratory processes. The sampling inlet of each instrument was positioned as close as possible to the suspected point of emission for a given process (indicated by arrows in Figure 1). We used an HHPC-6 hand-held particle counter (ART Instruments, Grants Pass, OR) to determine the airborne particle number concentration based on optical counting principles using laser light scattering. This instrument measured the total number of particles per liter (particles/L) of air across six specific size cut points: 300, 500, 1,000, 3,000, 5,000, and 10,000 nm. The second instrument used was a TSI model 3007 hand-held condensation particle counter (CPC; TSI, Inc., Shoreview, MN), operated as described by Methner (2008). The CPC unit measures particles in the size range of 10–1,000 nm, with data expressed as the total number of particles per cubic centimeter (particles/cc) of sampled air. The upper limit of detection for the HHPC-6 and CPC are 70,000 particles/L and 100,000 particles/cc, respectively. Because the size and degree of particle agglomeration were unknown at the time of this evaluation, we determined that using these particle-sizing instruments would provide a semiquantitative indication of the relative size range and magnitude of potential emissions for each process. Ambient/background particle number concentration measurements were collected inside each laboratory before each task/process and used to adjust the process-specific measurements via subtraction. Additionally, two general area air samples were collected before and after the laboratory processes at an area away from the processes, but in the same room, to serve as an indicator of background concentrations not related to specific processes.

### Transmission electron microscopy (TEM)

In addition to direct-reading instrumentation, filter-based air samples were collected to qualitatively determine whether engineered nanomaterials were emitted during the laboratory processes. The air sampling filters were positioned as close as possible to the suspected emission source (i.e., slightly above the analytical balance during weighing of material) (Figure 1) for the duration of the task or process to increase the probability of capturing nanomaterials and to simulate extreme case scenarios for laboratory personnel. This type of sampling strategy should not be interpreted as representative of full-shift worker exposure, yet it does provide an indication of potential worker exposure due to inadequate air sampling instrumentation that can be worn by workers to estimate CNMs in a worker’s personal breathing zone (Methner 2008). Sampling times ranged from 25 to 186 min (air volume, 175–1,300 L) and were dependent on the time necessary to complete the task being evaluated. The filter-based air samples were collected using Leland Legacy pumps (SKC Inc., Eighty Four, PA) that were operated at a sampling rate of 7.0 L/min. Pumps were calibrated before and after each day of sampling. Air samples were collected on 37-mm diameter, 0.8-μm pore size, open-face mixed cellulose ester membrane filters. Additionally, one general area air sample was collected at an area away from the process, but in the same room, to serve as an indicator of background concentrations not related to specific processes. Sample filters were then analyzed using TEM with energy dispersive spectroscopy and a digital image system for particle sizing and elemental composition. TEM allows the microscopist the ability to identify particles in the nanometer size range and the morphology of the particles (size, shape, degree of agglomeration). The sample filters were prepared by direct preparation in accordance with NIOSH Method 7402 (NIOSH 1994) using acetone vapor to collapse the filter media onto a copper TEM grid. A bulk sample of each material handled was deposited onto blank mixed cellulose ester filter media and prepped in a manner identical to other air samples. The bulk material was used by the microscopist to identify each nanomaterial of interest. At least 20 random grid openings per sample were examined via TEM. If the nanomaterial of interest was found, a digital image of the structure was captured. If no nanomaterial of interest was observed on the grids, the result for the sample was “none detected.”

## Results

### Airborne particle detection

**Figure 2:**
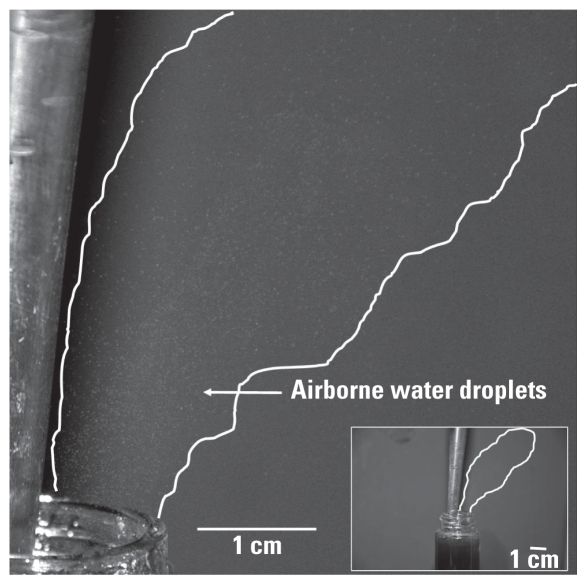
Aerosolization of water containing 100 mg/L NOM. Water droplets are visualized in a plume after sonication pulses (area between white lines). Inset: broader view of water droplet plume (indicated by white outline) after sonication pulse.

The goal of this study was to determine the potential for occupational exposure to CNMs when using environmentally relevant matrices to simulate environmental systems, such as streams, rivers, ponds, and reservoirs. These water bodies contain varying concentrations of NOM, a naturally acting surfactant that improves the aquatic suspension of hydrophobic chemicals such as organic pollutants and agglomerated CNMs (Chefetz and Xing 2009; Hyung et al. 2007; Kennedy et al. 2008). Figure 2 demonstrates that sonication of water containing 100 mg/L NOM resulted in the aerosolization of water droplets. This water droplet plume was generated during almost every sonication pulse. The cumulative effect over the course of the sonication process may result in substantial aerosolization of water droplets. This may be of concern when working with CNMs in NOM-containing waters because of the potential presence of CNMs in the water droplets.

The particle number concentrations measured for each of the eight CNMs and laboratory tasks/processes (i.e., weighing/handling CNMs and sonicating CNMs in aqueous suspensions) are presented in Table 1. After adjusting for background particle number concentrations, it was evident that increases in the airborne particle number concentration occurred during each process for almost all the CNMs examined. Airborne particle number concentrations were inversely related to particle size, with the size distribution of particles skewed toward those CNMs with an aerodynamic diameter < 1 μm. During handling of hydrophobic C60 and raw MWCNT, the highest airborne particle number concentrations were seen at the 300-nm size {53,119 particles/L for C60 and 123,403 particles/L for raw MWCNT [above the upper limit of detection (70,000 particles/L) for the HHPC-6], followed by the 500-nm size (3,884 particles/L for C60 and 34,446 particles/L for raw MWCNT)}. When analyzed at the 10–1,000 nm scale, airborne C60 and raw MWCNT particle number concentrations were higher than background particle number concentrations and approximately the same particle number concentrations (~ 1,500 particles/cc). Similar handling effects were seen by Maynard et al. (2004) when gentle air currents in the laboratory produced airborne single-walled carbon nanotube particles. Sonication caused aerosolization of C60 in a DI water suspension and raw MWCNT in a hard reconstituted water suspension containing 100 mg/L NOM. Sonication produced airborne C60 and MWCNT at concentrations approximately one-half and one-third, respectively, of those observed during the weighing process (23,856 particles/L for C60 and 42,796 particles/L for raw MWCNT in the 300-nm range). We observed a similar trend to the handling process during sonication, where highest particle number concentrations were in the 300- and 500-nm size ranges. However, sonication increased airborne C60 and raw MWCNT particle number concentrations in the 10–1,000 nm size range (2,176 and 2,776 particles/cc, respectively) compared with weighing and handling dry CNMs.

We observed a slightly different trend with MWCNT-OH and CB, two functionalized, water-soluble forms of CNMs. Airborne concentrations of MWCNT-OH and CB were very low during weighing and transferring, with the highest particle number concentrations detected in the 500-nm range (3,065 particles/L for MWCNT-OH and 1,428 particles/L for CB). This was confirmed in the 10–1,000 nm size range as well (676 and 0 particles/cc, respectively). However, sonication of MWCNT-OH in a moderately hard reconstituted water suspension with 100 mg/L NOM and CB in DI water suspension resulted in dramatically higher airborne particle number concentrations compared with handling dry CNMs. The highest particle number concentrations were in the 300-nm size range [144,623 particles/L for MWCNT-OH and 156,336 particles/L for CB; both of these values exceeded the upper limit of quantification of the HHPC-6 (70,000 particles/L), followed by the 500-nm range (65,402 particles/L for MWCNT-OH and 54,242 particles/L for CB]. In the 10–1,000 nm size range, there was no change in particle number concentrations between handling and sonicating MWCNT-OH, but there was an increase in particle number concentration when sonicating CB (1,057 particles/cc).

### TEM

**Figure 3:**
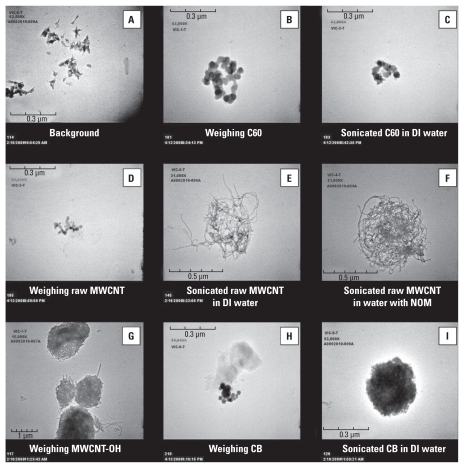
TEM images of engineered CNMs during laboratory processes. (*A*) Background air sample; bar = 0.3 μm. (*B*) Weighing/transferring C60 inside hood with no ventilation; bar = 0.3 μm. (*C*) Sonicating C60 in DI water inside unventilated enclosure; bar = 0.3 μm. (*D*) Weighing/transferring raw MWCNT inside hood with no ventilation; bar = 0.3 μm. Note that no tubular structures are present. (*E*) Sonicating raw MWCNT in DI water inside unventilated enclosure; bar = 0.5 μm. (*F*) Sonicating raw MWCNT in reconstituted water containing 100 mg/L (parts per million) NOM inside unventilated enclosure; bar = 0.5 μm. (*G*) Weighing/transferring MWCNT-OH inside hood with no ventilation; bar = 1 μm. (*H*) Weighing/transferring CB inside hood with no ventilation; bar = 0.3 μm. (*I*) Sonicating CB in DI water inside unventilated enclosure; bar = 0.3 μm.

Filter-based air samples were collected during each of the laboratory tasks and processes. TEM images verified the morphology and relative sizes of particles captured during the laboratory processes (Figure 3). All samples were collected as short-duration, process-specific area samples and were not in the breathing zone of the workers. The background sample image shows amorphous particles that were identified as not being engineered CNMs (Figure 3A). C60 particles were agglomerated during handling but partially deagglomerated when sonicated (Figure 3B and C, respectively). Figure 3D, E, and F represent raw airborne MWCNT during weighing, sonication in DI water, and sonication in moderately hard reconstituted water containing 100 mg/L NOM, respectively. Note that typical tubular structures are missing from raw MWCNT during the handling process. However, we observed tubular structures during sonication in both types of suspension, with more tubes aerosolized and captured on the filter when in water containing NOM. The raw MWCNT agglomerates featured in the TEM images for both suspensions were approximately 500 nm in diameter. MWCNT-OH was highly agglomerated when handled, with a diameter of > 1,000 nm (Figure 3G). Airborne CB was somewhat agglomerated during handling and more highly agglomerated when sonicated in DI water (Figure 3H and I, respectively).

## Discussion

This case study served as a range-finding survey of airborne nanomaterials emitted during common tasks performed in a laboratory that investigates environmental risks of engineered nanomaterials. In addition, this study allowed the NIOSH NTRC field team to test analytical equipment and methodologies under various laboratory conditions to evaluate potential occupational exposure to engineered nanomaterials. Specifically, this research effort combined semiquantitative airborne particle number concentrations with qualitative TEM imaging to provide a weight-of-evidence evaluation of whether engineered nanomaterials were released during laboratory tasks. To our knowledge, this is the first study to suggest that engineered nanoparticles may be released from aqueous suspensions during sonication. Results of this study imply that the commonly held belief that engineered nanomaterials in suspension during sonication pose low risk of inhalation exposure may need some reconsideration. This is especially true with regard to results from a recent international survey of nanomaterial firms and laboratories; in that study, Conti et al. (2008) found that many workers in the field think nanomaterials pose no risk.

After accounting for background particle counts, we detected increased particle number concentrations during the handling of dry CNMs and also during the sonication of CNM suspensions (Table 1). An interesting observation during the present study was the differential behavior between hydrophobic and hydrophilic CNMs with regard to different laboratory processes. During material handling and weighing, we observed higher airborne particle number concentrations of the hydrophobic CNMs (C60 and raw MWCNT) compared with hydrophilic CNMs. Lower particle number concentrations of aerosolized CNMs at 300 and 500 nm were noted during sonication, yet cumulative particle number concentrations in the 10–1,000 nm size range were elevated compared with the handling process. This finding was more pronounced when raw MWCNTs were sonicated in moderately hard reconstituted water containing 100 mg/L NOM, suggesting that sonication of CNM suspensions may increase the number of smaller-sized CNM agglomerates (i.e., < 300 nm)—as would be expected with sonication—that were not detected by the HHPC-6 particle counter. An opposite pattern was observed when the hydrophilic CNMs (MWCNT-OH and CB) were compared. Very low particle number concentrations were detected during handling of hydrophilic CNMs, yet sonicating these hydrophilic CNMs, whether in a DI water suspension or a moderately hard reconstituted water suspension with 100 mg/L NOM, resulted in dramatically higher airborne particle number concentrations. From this finding, along with visual evidence provided by TEM examination of the air-sampling filters, we hypothesize that CNM agglomerates are being emitted to the laboratory atmosphere in water droplets. These data demonstrate that care should be exercised when handling dry hydrophobic CNMs and also when sonicating wet CNMs in suspension. A similar pattern of emissions and potential exposure was observed by Methner et al. (2007) during a study of nanomaterial polymer laboratory workers.

In the present study, all filter-based air samples collected during weighing and transfer processes, with the exception of raw MWCNT, showed the presence of the engineered nanomaterial handled. Likewise, all samples collected during sonication, regardless of the nanomaterial in suspension, showed visual evidence of the presence of the engineered nanomaterial when analyzed by TEM. The majority of the images presented in Figure 3 indicate that single spheres or nanotubes are more the exception than the rule; most particles showed clear evidence of agglomeration. However, this may be due to the current methodology that uses 0.8-μm filter membranes, which may allow small, individual CNMs to pass through and thus be unavailable for analysis. The images shown in Figure 3 clearly provide strong visual evidence that emissions from specific tasks and processes can occur. No evidence of engineered nanomaterial was present on the background air filter sample collected.

**Figure 4:**
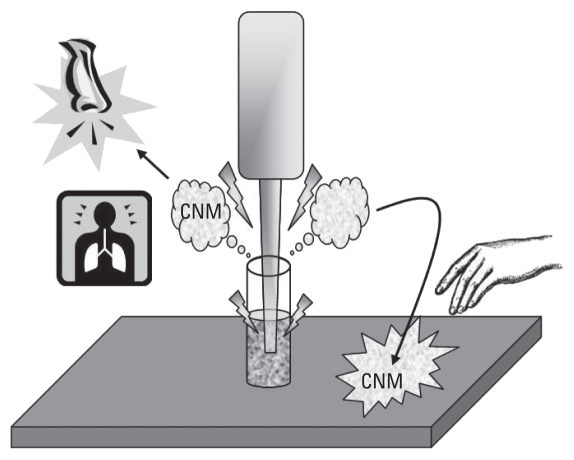
Graphical representation of potential exposure to engineered CNMs in the laboratory through inhalation and dermal contact.

Our data indicate that although suspensions may minimize aerosolization of CNMs relative to their dry form, sonication of such suspensions outside protective enclosures can result in aerosolization and thus potential exposure to nanosized particulates (Figure 4). If sonication occurs outside an enclosure, as often occurs in laboratory settings, the proximity of the researcher’s breathing zone may result in inhalation of CNM particulates in water droplets and/or mists. Similarly, airborne water droplets can be generated by standard aquaria that use air stones or other air supplies to aerate test waters during long-term aquatic toxicology studies. The airborne CNMs in water droplets have the potential to cause pulmonary effects similar to those described for particulate matter, single-walled carbon nanotubes (SWCNT), and MWCNTs (Laks et al. 2008; Lam et al. 2006; Ma-Hock et al. 2009; Mitchell et al. 2007; Shvedova et al. 2008). The mass balance of CNMs collected during the laboratory processes were not determined, so the mass of airborne CNMs is unknown, making it difficult to compare with CNM inhalation toxicity studies or to occupational exposure limits for carbon-based materials such as respirable graphite or particulate matter. However, Conti et al. (2008) found that organizations that used nanomaterials in suspensions or embedded in matrices were less likely to make recommendations for respiratory protection. With sonication being a critical component of nanomaterial synthesis and deagglomeration, this survey result suggests that inhalation exposure may be an overlooked safety component during this commonly used laboratory process.

Despite being housed in an enclosure during this experimental process evaluation, the sonicator has the potential to emit engineered nanomaterials when the enclosure door is opened after the sonication process is complete. If this occurs, airborne CNMs may be inhaled by laboratory workers. In addition, if the sealing gasket around the perimeter of the enclosure door is damaged or otherwise breached, release of aerosolized droplets to the laboratory atmosphere may result. Furthermore, these airborne CNM-containing water droplets have the potential to deposit on other surfaces within the sonication cabinet and in the laboratory. Once dried, CNM may become resuspended if disturbed and potentially result in exposure via inhalation. Finally, these nanomaterials may be available for dermal deposition if laboratory workers unknowingly contact contaminated surfaces with unprotected skin (e.g., hands, forearms). Currently, there are no occupational exposure limits specific to engineered nanomaterials (Methner 2008); however, basic precautionary procedures and control equipment can dramatically reduce airborne releases of nanomaterials (NIOSH 2009). Therefore, environmental scientists should implement a general or nano-specific environmental, health, and safety program at their organizations (Conti et al. 2008), use personal protective equipment, and develop standard operational procedures to minimize potential hazards when working with engineered nanomaterials in environmentally relevant laboratory systems.

Although this preliminary research has generated some interesting and relevant findings, specific uncertainties associated with experimental design and implementation need to be addressed. First, this single-case study was designed to determine the relative magnitude of airborne nanomaterial emissions associated with tasks and materials used in environmental laboratory experiments. We used only a single data point for each of the tasks and materials during this first assessment. Thus, the data presented here are not statistically based. These data should be viewed as an indicator of the need for additional studies that focus on a robust statistically based experimental design, experimental variables, specific engineered nanomaterials, and sample collection. Second, the data interpretation can be confounding because of the two different particle counters used to measure airborne nano-sized particles. The two particle counters use different counting principles, counting efficiencies, and size ranges, so the data are not directly convertible to identical units. Our intentions were to show the size ranges and relative number concentrations on a task- specific basis. This way, a reader can examine the data separately according to task and determine which task emitted nanomaterials. Thus, these data should be interpreted as relative indicators of CNM release, especially since the data were adjusted by subtracting background particle number concentrations. Furthermore, because the direct-reading, real-time instrumentations are not material specific (e.g., MWCNTs or CB only) and cannot identify the chemical composition of the particles detected (e.g., MWCNT vs. background particulate matter or water droplets), we cannot definitively conclude that increases in particle number concentrations for a specific operation are due to a release of particulate material from that process. However, because the particle number concentrations in the lower size ranges were higher than background and the results of the TEM analyses yielded visual evidence of the engineered nanomaterials, we can conclude that a release occurred and that the potential for exposure exists.

## Conclusions

Care must be taken when conducting laboratory studies using CNMs in environmentally relevant matrices. Sonicating hydrophobic CNMs in DI water suspensions results in airborne particle number concentrations lower than when handling dry CNMs. In contrast, sonicating hydrophilic CNMs in a moderately hard reconstituted water suspension containing natural surfactants dramatically increases airborne CNM particles compared with handling of dry CNMs. Thus, researchers using these environmentally relevant matrices should use appropriate protective equipment (respiratory and dermal protection) in addition to employing adequate engineering controls to minimize CNM aerosolization during preparation and experimental usage. Although we examined laboratory processes in an environmental research laboratory, similar results are also possible in other laboratories that use similar materials (e.g., functionalized CNMs), similar tasks (e.g., sonication), and similar dispersive agents (e.g., surfactants). Additional research is needed to better characterize CNM emissions and worker exposure during handling and sonication to corroborate the results of this case study.

## Figures and Tables

**Table 1:** Airborne particle number concentrations emitted during laboratory processes.

Task/sample location	Particle size range (nm)	Measured particle number concentration	Average^a^ background particle number concentration	Adjusted particle number concentration^b^
Weighing C_60_ fullerenes and transfering to mixing beaker inside hood with ventilation off	300^c^	66,813	13,694	53,119
	500	4,875	991	3,884
	1,000	338	176	162
	3,000	59	56	3
	5,000	0	5	0
	10,000	0	0	0
	10–1,000^d^	2,200	724	1,476

Sonication of C_60_ fullerenes in DI water	300	37,550	13,694	23,856
	500	7,492	991	6,501
	1,000	1,067	176	891
	3,000	109	56	53
	5,000	3	5	0
	10,000	0	0	0
	10–1,000	2,900	724	2,176

Weighing raw MWCNT and transfering to mixing beaker inside hood with ventilation off	300	137,097^e^	13,694	123,403^e^
	500	35,437	991	34,446
	1,000	4,514	176	4,338
	3,000	106	56	50
	5,000	1	5	0
	10,000	0	0	0
	10–1,000	2,300	724	1,576

Sonication of raw MWCNT in reconstituted water containing 100 mg/L NOM	300	56,490	13,694	42,796
	500	24,768	991	23,777
	1,000	2,360	176	2,184
	3,000	142	56	86
	5,000	0	5	0
	10,000	0	0	0
	10–1,000	3,500	724	2,776

Weighing functionalized MWCNT and transfering to mixing beaker inside hood with ventilation off	300	12,851	13,694	0
	500	4,056	991	3,065
	1,000	1,875	176	1,699
	3,000	336	56	280
	5,000	9	5	4
	10,000	0	0	0
	10–1,000	1,400	724	676

Sonication of functionalized MWCNT in reconstituted water containing 100 mg/L NOM	300	158,317^e^	13,694	144,623^e^
	500	66,393	991	65,402
	1,000	6,381	176	6,205
	3,000	52	56	0
	5,000	0	5	0
	10,000	0	0	0
	10–1,000	1,450	724	726

Weighing CB and transfering to mixing beaker inside hood with ventilation off^f^	300	9,775	9,204	571
	500	2,012	584	1,428
	1,000	1,169	144	1,025
	3,000	445	52	393
	5,000	86	3	83
	10,000	50	0	50
	10–1,000	660	1,250	0

Sonication of CB in DI water^f^	300	165,540^e^	9,204	156,336^e^
	500	54,826	584	54,242
	1,000	7,121	144	6,977
	3,000	336	52	284
	5,000	1	3	0
	10,000	0	0	0
	10–1,000	2,307	1,250	1,057

a Average background number concentration was computed from two measurements obtained inside the room before material handling began and two measurements obtained after handling ceased.

b If the difference between the measured particle number concentration and the average background particle number concentration was less than zero, the adjusted particle number concentration was reported as zero.

c Particles in the range of 300–10,000 nm were quantified with the HHPC, and particle concentrations are given as particles/L.

d Particles in the 10–1,000 nm range were quantified with the CPC, and particle concentrations are given as particles/cc.

e Particle counts exceed the upper limit of quantification for the HHPC (70,000 P/L) or the CPC (100,000 P/cc).

f Because of a change in background particle number concentration, a new average background particle number concentration was calculated for these tasks.
